# Evidence for disaster risk reduction, planning and response: design of the Evidence Aid survey

**DOI:** 10.1371/currents.RRN1270

**Published:** 2011-10-14

**Authors:** Mike Clarke, Bonnix Kayabu

**Affiliations:** ^*^Director, All Ireland Hub for Trials Methodology Research; Evidence Aid and ^†^Evidence Aid Coordinator, Centre for Global Health, Trinity College Dublin, Ireland

## Abstract

Systematic reviews are now regarded as a key component of the decision making process in health care, and, increasingly, in other areas. This should also be true in disaster risk reduction, planning and response. Since the Indian Ocean tsunami in 2004, The Cochrane Collaboration and others have been working together to strengthen the use and usefulness of systematic reviews in this field, through Evidence Aid. Evidence Aid is conducting a survey to identify the attitudes of those involved in the humanitarian response to natural disasters and other crises towards systematic reviews and research in such settings; their priorities for evidence, and their preferences for how the information should be made accessible. This article contains an outline of the survey instrument, which is available in full from www.EvidenceAid.org. The preliminary findings of the survey will be published in future articles.

## Background

The role of systematic reviews in health [1] [2] and other areas [3] is increasingly well established. Since the Indian Ocean tsunami in 2004, The Cochrane Collaboration and others have been working together to strengthen the use and usefulness of systematic reviews for people working in disaster risk reduction, planning and response, through Evidence Aid. [4] [5] [6] This involved the release in March 2011 of special collections of Cochrane reviews on fracture management, safe water and post-traumatic stress disorder in response to the earthquake and tsunami in Japan; these were also translated into Japanese and made available on the internet. 

## Purpose of the needs assessment survey

Evidence Aid is now conducting a survey to identify the attitudes of those involved in the humanitarian response to natural disasters and other crises towards systematic reviews and research in such settings; their priorities for evidence, and their preferences for how the information should be made accessible. This article contains an outline of the survey instrument, which is available in Arabic, French and Spanish, as well as English (www.EvidenceAid.org). Anyone involved in the disaster field, or working in humanitarian relief more generally, is welcome to complete the online survey or to request an electronic or paper version from the authors. The wider the response, the more useful the findings will be for developing new knowledge resources and improving existing ones. In keeping with the rapid publication model for PLoS Currents: Disasters, future articles will present the accumulating findings from the survey, leading to the final report and the plans to respond to these findings. 

 The Evidence Aid needs assessment survey aims to identify the challenges that aid agencies face when they need up-to-date knowledge to plan and respond to natural disasters and other humanitarian emergencies. [7] The potential role of systematic reviews to help with this is being assessed broadly using mixed methods, including a combination of quantitative and qualitative questions. It is expected that the qualitative questions will reveal the challenges to following best practice in the humanitarian sector, with participants being asked to provide their experiences of using systematic reviews and other sources of knowledge when making decisions. The quantitative questions should provide estimates of the size of the need and the most preferred options for meeting this need.

## Development of the needs assessment survey

The survey was developed following a formal evaluation of Evidence Aid in 2008/09 [6] and subsequent discussions with people working in a variety of aid agencies. These people are connected with a variety of organisations and work in a variety of disciplines including health, nutrition, water and sanitation, displaced people, etc. They were identified through existing connections for Evidence Aid and suggestions during conference calls and face-to-face meetings. Additional contacts were established through searches of the websites for aid agencies. These discussions helped to raise awareness of Evidence Aid and allowed a discussion of the role of research evidence in decision making. These preliminary contacts also helped with the design of the survey. 

 A small group of these contacts and colleagues involved in Evidence Aid, systematic reviews or humanitarian aid piloted the survey, leading to further changes and refinements to the online English version, before this was opened in July 2011. The finalised English version has also been translated into Arabic, French and Spanish recognising the language barrier for many people and organisation involved in disasters or humanitarian relief. This is likely to provide rich information from aid workers in different regions of the world, whose culture and aid agencies’ agenda might differ dramatically from those of anglophone organisations. 

The survey is provided at the end of this article. It has three sections. The first section collects demographic information on the respondent, section 2 is to be completed by those who work in disasters or the humanitarian area more generally, and section 3 is for those working in agencies that provide funding for the delivery of humanitarian relief. Participants who fit the criteria for both section 2 and section 3 are asked to answer the questions in both these sections.

## Promotion of the needs assessment survey

Information about the survey was sent to contacts established during the aforementioned discussions, and a snowballing technique was used to cascade knowledge to others. This included distribution through the Information Services of the World Health Organisation. This initial information included the url for the online survey in Survey Monkey and efforts are underway to include this link on the websites of relevant agencies. Future distribution of the survey will include the availability of a document that can be downloaded and completed either electronically or on paper. 

## Analysis of the needs assessment survey

The survey contains a mixture of picklist items and open questions, with the ability to provide comments for questions as appropriate. The responses are being monitored online. Data analyses will combine the preparation of descriptive statistics and analyses of correlation between respondent characteristics and their answers, for the quantitative data. This will be done partially through the analyses options in Survey Monkey and SPSS. The qualitative responses will be analysed using NVivo and free text responses will also be used in a narrative analysis of, for example, the priorities for evidence. 

## Dissemination of the findings

The findings of the survey will be sent to all participants who request this. Interim analyses were presented at the Evidence Aid Conference in Oxford on September 26 2011 and will be made available in rapid, open access publications to stimulate debate and encourage further responses. Summary reports will also be made available for dissemination through participating aid agencies and other partners in Evidence Aid. The findings will inform the development of the future strategy for Evidence Aid, by refining its purpose and intended audience, identifying ways to increase awareness in the humanitarian aid community, improving the coverage of relevant topics, and improving the relevance of the findings of systematic reviews to decision makers (for example by summarizing multiple reviews or adding contextual information). [6]   

## Conclusions

Since Evidence Aid was initiated by the Cochrane Collaboration after the Indian Ocean tsunami, it has been actively engaged in the provision of timely knowledge to people involved in disaster risk reduction, planning and response throughout the world. The initial focus was on healthcare interventions but this is now being expanded. This needs assessment survey is a key step to understanding the real needs of aid agencies and others about access to reliable, timely, up-to-date knowledge and in building partnerships between the producers and users of evidence. The findings of the survey should encourage the use of systematic reviews by decision makers in the humanitarian sector, providing them with credible information when they are facing uncertainties and making choices. It should help Evidence Aid achieve its aim of providing high quality, accessible evidence on which interventions work, don’t work and are unproven, in an effective and timely way; partnering with those who need the evidence, those who produce it and those who translate the knowledge on the ground, to improve outcomes for people and communities. 

## Acknowledgements

We are grateful to everyone who has helped in the design and implementation of the survey, in particular Tari Turner who conducted the earlier evaluation of Evidence Aid,[6] Sara Sentis from the Iberoamerican Cochrane Centre for the Spanish translation, Amani Alhajeri from the Bahrain Branch of the UK Cochrane Centre for the Arabic translation, Kimberley Parker and Jonathan Abrahams at the World Health Organisation for their help with the distribution of the survey and Muireann Brennan from CDC for her advice during the early stage of the design of survey.   

### Funding Information

Work on the Evidence Aid survey was made possible by grants from The Cochrane Collaboration and Wiley-Blackwell, to the Centre for Global Health in Trinity College Dublin, Ireland; and by in-kind support from the Centre for Public Health, Queen's University Belfast, Northern Ireland.

### Competing Interests

The authors have no known competing interests, other than their involvement in Evidence Aid, the future of which is likely to be determined, at least in part, by the findings of this needs assessment survey.

### Items being collected in the needs assessment survey

(Full version is available from www.EvidenceAid.org)

#### 1. Demographic information on all respondents

1.01 Name (optional)  

1.02 Degrees or qualifications  1.03 Country  1.04 Profession  1.05 Current types of activity  1.06 Current job  1.07 Organizations worked with in humanitarian crises  1.08 Whether these organizations are active in the humanitarian sector or related areas, provide funding, or both

#### 2. Questions for humanitarian workers 

2.01 Duration of experience in disaster planning, response or both  

2.02, 2.03 Types of emergency worked in (natural disasters, conflict settings, other)  2.04.1 Types of disaster worked in  2.04.2 Countries worked in  2.04.3 Types of intervention involved with  2.04.4 Role in this work  2.05 Knowledge of Cochrane reviews or other systematic reviews  2.06 Preference for the presentation of the findings of systematic reviews (whole review, whole review plus comments from experts in the humanitarian sector, review summary on its own, summary and context specific information)  2.07 Preference for access to the findings of systematic reviews (full systematic reviews or summaries of systematic reviews; online, email, CD or DVD, mobile technology, paper)  2.08, 2.09, 2.10 Experience of using systematic reviews in decision making  2.11 Opinion on the usefulness of systematic reviews for disasters  2.12 Opinion on the role of evidence from scientific research in informing policy  2.13 Opinion on the need to supplement evidence prepared for a general audience with local evidence  2.14 Experience of the use of evidence for policy  2.15 Opinion on the relative importance of anecdotal evidence, intuition, personal experience, scientific evidence, cultural norms and organizational usual practice  2.16, 2.17 Three (or more) priority topics for evidence in natural disasters or other humanitarian crises  2.18 Opinion on the timing of the provision of evidence from systematic reviews (when a natural disaster is not known to be imminent; during the period of prediction that a disaster will happen; during and shortly after a disaster, after a natural disaster)  2.19, 2.20 Impact of the findings of systematic reviews on the implementation of interventions  2.21 Need for training in doing systematic reviews  2.22, 2.23 Opinion on the potential impact of improved access to systematic reviews in natural disasters and other humanitarian crises  2.24, 2.25 Barriers to the use of systematic reviews  2.26, 2.27 Opinion on the role of evidence and systematic reviews in choosing humanitarian interventions  2.28, 2.29 Preference for the source of research evidence  2.30 Opinion on the organization’s attitude to evidence-based practices  2.31, 2.32 Opinion on the organization’s attitude to organizational learning  2.33, 2.34 Opinion on attitudes of donors towards systematic reviews  2.35 Use of evidence in reports to donors  2.36, 2.37, 2.38 Sources of knowledge used to support decisions  2.39, 2.40, 2.41, 2.42 Knowledge and use of the SPHERE handbook  2.43, 2.44, 2.45, 2.46 Knowledge and use of the International Disaster Database (EM-DAT) project  2.47 Use of other guidelines  2.48, 2.49 Opinion on the conduct of clinical trials of interventions in disaster settings

### 3. Questions for donor organizations

3.01, 3.02 Knowledge of Cochrane reviews or other systematic reviews  

3.03 Opinion on the use of systematic reviews in natural disasters and other humanitarian crises  3.04, 3.05 Use of systematic reviews in natural disasters or other humanitarian crises  3.06 Types of project funded in disasters and other humanitarian crises  3.07 Methods used to evaluate the impact of humanitarian projects  3.08 Methods used to ensure that agencies implement cost effective interventions  3.09 Opinion on the organization’s attitude to evidence-based practice  3.10 Opinion on the relative importance of anecdotal evidence, intuition, personal experience, scientific evidence, culture norms and organizational usual practice  3.11 Opinion on the organization’s attitude to organizational learning  3.12 Opinion on the role of systematic reviews in assessing the likely effects of projects  3.13 Organization’s use of systematic reviews to assess the likely impact of projects  3.14, 3.15 Key factors considered before providing funding to a relief agency  3.16 Topics for which research evidence would help when making decisions about funding a project  3.17 Topics for which research evidence would help when analyzing reports from recipient agencies  3.18, 3.19 Preference for access to the findings of systematic reviews (full systematic reviews or summaries of systematic reviews; online, email, CD or DVD, mobile technology, paper)  3.20, 3.21 Opinion on the need for agencies to use systematic reviews when planning for or responding to emergencies3.22 Opinion on the role of evidence-based interventions and the use of systematic reviews in emergency contexts  3.23, 3.24 Opinion on the conduct of clinical trials of interventions in disaster settings
